# HCV Treatment Outcomes in PWID: Impact of Addiction History on SVR12

**DOI:** 10.3390/microorganisms12122554

**Published:** 2024-12-11

**Authors:** Ivana Milošević, Branko Beronja, Ana Filipović, Nikola Mitrović, Jelena Simić, Nataša Knežević, Jovana Ranin, Nevena Todorović, Olja Stevanović, Aleksandra Radovanović-Spurnić, Nataša Katanić, Dejan Hristović, Nataša Nikolić

**Affiliations:** 1 Clinic for Infectious and Tropical Diseases, University Clinical Center of Serbia, Bulevar Oslobođenja 16, 11000 Belgrade, Serbia; anafilipovic0211@gmail.com (A.F.); nikola.mitrovic@med.bg.ac.rs (N.M.); simicj093@gmail.com (J.S.); natasaknezevic995@gmail.com (N.K.); jovana.ranin@hotmail.rs (J.R.); nevena.todorovic.1992@gmail.com (N.T.); stevanovicolja74@gmail.com (O.S.); aleksandra.spurnic@med.bg.ac.rs (A.R.-S.); katanicn@gmail.com (N.K.); 2 Faculty of Medicine, University of Belgrade, Dr Subotica 8, 11000 Belgrade, Serbia; brankoberonj99@gmail.com; 3 Department of Infective Diseases, Faculty of Medicine, University of Pristina Temporarily Settled in Kosovska Mitrovica, 38220 Kosovska Mitrovica, Serbia; 4 Clinic for Infectious and Tropical Diseases, Military Academy of the University of Defence, Crnotravska 17, 11000 Belgrade, Serbia; dhristovic@gmail.com

**Keywords:** chronic hepatitis C, SVR12, people who inject drugs, PWID, follow-up

## Abstract

People who inject drugs (PWIDs) experience high rates of hepatitis C virus (HCV) infection, primarily due to needle sharing and limited healthcare access, resulting in a disproportionate disease burden within this population. This prospective study evaluated treatment outcomes in 432 adult patients with chronic hepatitis C (CHC) treated with direct-acting antivirals (DAAs) at the University Clinical Center of Serbia. Patients were categorized into two groups based on a history of drug addiction: PWIDs (163, 37.7%) and non-PWIDs (269, 62.3%). The PWID group was further categorized into subpopulations of problematic PWIDs (39, 23.9%), ex-PWIDs (124, 76.1%), and PWIDs on OST (96, 58.9%). The PWID group demonstrated significantly lower treatment adherence, with an intention-to-treat (ITT) rate of 82.8%, compared to 96.3% in the control group (*p* < 0.001). In contrast, no significant differences were observed in per-protocol (PP) outcomes between the two groups. Additionally, PWIDs were significantly younger (*p* < 0.001) and had higher rates of psychiatric disorders (*p* < 0.001), alcohol abuse (*p* < 0.001), and HCV genotype 1a (*p* < 0.001). Advanced fibrosis was predictor of PP treatment failure among PWIDs, while mood disorders and alcohol use disorder were associated with interruptions before the scheduled completion time. For non-PWIDs, older age and advanced fibrosis emerged as key predictors of PP treatment failure. The loss to follow-up was most commonly observed in the problematic PWID subgroup (*p* = 0.001). These findings highlight the importance of addressing barriers in PWIDs through integrated care strategies that concurrently manage addiction and HCV.

## 1. Introduction

PWIDs are among the populations most heavily affected by HCV, with high infection rates driven by factors like needle sharing and limited access to healthcare services [1]. HCV seroprevalence among PWIDs varies significantly based on geographic location, with estimates ranging from 18% to 88% [2]. According to the World Health Organization (WHO), at least 39.4% of PWIDs are living with active HCV infection, highlighting the high burden of viremic HCV in this population [3]. In the absence of treatment and viral clearance, PWIDs with CHC are likely to develop progressively severe liver complications, including hepatocellular carcinoma (HCC), as they reach middle to late adulthood [4]. Although treatment for PWIDs is essential for elimination of CHC as a public health issue, there remains a certain reluctance to implement DAAs within this population [5]. This hesitance can be attributed to factors such as stigma, concerns about adherence, and ongoing substance use, which complicate treatment dynamics and can impact overall outcomes [6]. Treating PWIDs is crucial for reducing overall HCV transmission in communities, making it a priority for public health efforts aimed at HCV elimination, according to leading health organizations, including the World Health Organization (WHO), the American Association for the Study of Liver Diseases (AASLD), and the European Association for the Study of the Liver (EASL) [1,5,6,7,8]. The aim of the study was to establish potential differences between PWIDs and other patients with CHC. The primary objective was to analyze whether current or previous addiction affects the SVR12 rate. The secondary objective was to establish predictors for achieving SVR12 among PWIDs and other patients with CHC.

## 2. Materials and Methods

**Figure 1:**
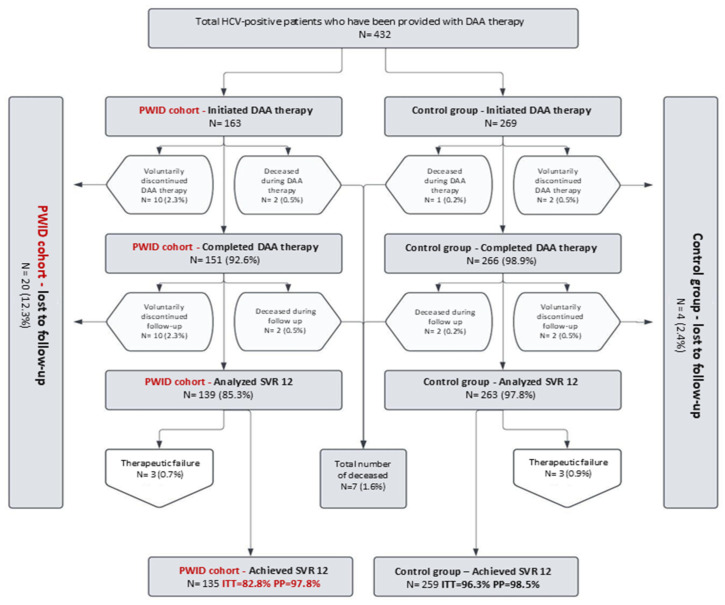
Flowchart of the study, focusing on follow-up points. Abbreviations: ITT—Intention to Treat, PP—Per Protocol.

This prospective study was conducted in Clinic for Infectious and Tropical Diseases at University Clinical Center of Serbia in Belgrade. The study included 432 adult patients diagnosed with chronic hepatitis C (CHC) who were treated with direct-acting antivirals (DAAs) between August 2022 and August 2024. The patients were categorized into two groups: 163 (37.7%) individuals with a history of previous or current addiction, and a control group comprising the remaining 269 (62.3%) treated patients. The graphical representation of the participant follow-up is provided in Figure 1.

The diagnosis of chronic hepatitis C (CHC) was confirmed in patients who tested positive for anti-HCV antibodies by detecting HCV RNA in the blood for a minimum of six months [9].

All patients underwent liver fibrosis assessment by non-invasive methods—liver stiffness measurement (FibroScan^®^, Miami, FL, USA) or fibrosis-4 (FIB-4) index [10]. A liver biopsy was performed in a small number of patients who were not eligible for liver stiffness measurement or FIB-4 assessment due to excessive weight, elevated transaminases, or combined liver disease etiology. Therefore, the liver fibrosis stage was determined by pathohistological examination of liver tissue obtained through biopsy.

Genotyping was performed using the cobas^®^ GT HCV genotyping test (Roche Diagnostics, Sandhofer Strasse 116, Mannheim, Germany) and the Abbott RealTime HCV Genotype II assay (Abbot GmbH, Max-Planck-Ring2, Wiesbaden, Germany) [11].

Patients were treated with direct-acting antivirals (DAAs) following the 2020 European Association for the Study of the Liver (EASL) guidelines. [6]. The selection of DAA depended on genotype, degree of fibrosis, and potential interactions between DAAs and other medications that patients were regularly taking for comorbidities. Elbasvir/grazoprevir (EBR/GZR) was the drug of choice for genotype 1b (GT1b) infection, while pangenotypic regimens, including glecaprevir/pibrentasvir (G/P) and sofosbuvir/velpatasvir ± ribavirin (SOF/VEL ± RBV), were utilized for all other genotypes. Drug–drug interactions (DDIs) were assessed using the Hep Drug Interaction tool provided by the University of Liverpool [12]. HIV co-infection did not influence the choice of DAA therapy. Ribavirin (RBV) was added to SOF/VEL in cases of cirrhosis and genotype 3. The duration of therapy with G/P was extended from 8 to 12 weeks for treatment-experienced patients and up to 16 weeks for treatment-experienced patients with cirrhosis and genotype 3. The success of the therapy, defined as a stable virological response, was characterized by a negative HCV RNA result 12 weeks post-treatment completion (SVR12).

In this study, the PWID population was categorized into ex-PWIDs (124, 76.1%), problematic PWIDs (39, 23.9%), and PWIDs on OST (96, 58.9%) to account for differences in drug use patterns and treatment needs, which may affect SVR12 outcomes. Ex-PWIDs are individuals who have not used drugs for over 2 years and have maintained stable abstinence for at least two years. Problematic PWIDs are active injectors and/or with a history of unstable abstinence, as documented in psychiatric evaluations. PWIDs on OST are those receiving opioid substitution therapy. It is important to note that there is overlap between the PWIDs on OST category and the other two groups. Within the PWIDs on OST group, significantly more participants were classified as ex-PWIDs (85, 88.5%) compared to problematic PWIDs (11, 11.5%) (*p* = 0.001). This classification allows for more precise analysis by considering the different risks and treatment requirements within these subgroups.

In evaluating the effectiveness of DAAs drugs for treating HCV, intention-to-treat (ITT) and per protocol (PP) approaches are used, each with distinct purposes. The ITT approach includes all patients who started treatment, regardless of protocol adherence, offering a realistic assessment of treatment effectiveness within the clinical population by accounting for potential therapy discontinuations. In contrast, the PP analysis includes only patients who fully completed treatment as per the protocol, allowing an estimation of the drug’s maximal efficacy under optimal conditions. By combining ITT and PP approaches to analyze SVR12, researchers gain insights into both real-world clinical outcomes and the drug’s potential in ideal circumstances, thus supporting the refinement of therapeutic strategies for HCV.

The study was conducted in accordance with ethical standards outlined in the Declaration of Helsinki [13]. Patients provided written consent for the use of their data related to disease and treatment for the purposes of this study. No personal identifiable information was used or compromised. Ethical approval was also obtained from the Ethics Committee of the University Clinical Center of Serbia (No 307/14).

The analytical approach employed descriptive and inferential statistical methods using IBM SPSS Statistics software, version 25.1 (IBM Corp., Armonk, NY, USA), with statistical significance determined at a threshold of *p* < 0.05. Continuous variables were summarized by means and standard deviations, whereas categorical variables were represented by frequencies and percentages. The distribution normality of continuous variables was evaluated using the Kolmogorov–Smirnov test. For assessing differences in laboratory parameters pre- and post-treatment, the non-parametric Wilcoxon signed-rank test was utilized, given its suitability for paired sample analyses.

The Cox proportional hazards model was implemented to investigate predictors of SVR12 achievement across two patient cohorts—intention-to-treat (ITT) and per-protocol (PP). In the Cox model, the time component was defined as the interval from treatment initiation to either SVR12 assessment or patient attrition. Initially, a univariate analysis was performed on all variables, with those demonstrating marginal signify (*p* < 0.125) subsequently entered the multivariate model. To ensure model validity and mitigate the risk of overfitting, variables were organized into three models: the first incorporated comorbidities, co-infections, and lifestyle variables; the second included liver fibrosis stage; and the third was focused on laboratory parameters. A fourth model was constructed specifically to evaluate the duration of abstinence and supplementary therapy in PWID patients, in accordance with the ITT/PP approach. Each model was adjusted for sex, age, and liver fibrosis stage to account for confounding effects, thereby enhancing the rigor and reliability of the findings.

## 3. Results

This study included a total of 432 participants, categorized into two groups based on a history of intravenous drug use: PWIDs (163, 37.7%) and non-PWIDs (269, 62.3%). The PWID group comprised a significantly higher proportion of male participants (76.7%, *p* < 0.001), whereas gender distribution in the non-PWID group was nearly equal (50.6% males, 49.4% females). No statistically significant age differences were observed between genders within either group (*p* = 0.254 for PWIDs, *p* = 0.595 for non-PWIDs). The average age of participants was 54.32 ± 10.25 years, with PWIDs averaging 45.74 ± 8.81 years and non-PWIDs 57.49 ± 14.57 years, showing a significantly higher age in the non-PWID group (*p* < 0.001). Self-initiated discontinuation of DAA therapy and/or follow-up was significantly more frequent in the PWID group (12.3% vs. 2.4%, *p* < 0.001). Medication adherence in both study groups classified under the per-protocol category was measured at 95%. No statistically significant difference in achieving SVR12 was observed between PWIDs and non-PWIDs who were treated and monitored according to the protocol (97.8% vs. 98.5%, *p* = 0.258). During the study, seven (1.6%) patients died at various stages of treatment and follow-up.

The PWID group had higher rates of neuroses and psychoses (*p* < 0.001), mood disorders (*p* < 0.001), alcohol use disorder (*p* = 0.001), and HIV coinfection (*p* = 0.021). In contrast, the non-PWID group had different comorbidities, such as hypertension (*p* < 0.001), diabetes (*p* = 0.003), chronic kidney disease (*p* = 0.014), and malignancies (*p* < 0.001). The distribution of other comorbidities is detailed in Table 1. In the PWID group, the highest number of patients were in abstinence for 1 to 10 years (113, 69.3%) and without opioid substitution therapy (67, 41.1%).

The laboratory parameters measured immediately before the initiation of treatment and 12 weeks after its completion are presented in Supplementary Table S1. The analyzed drug–drug interactions (DDIs) between DAAs and other medications used in chronic therapy are presented graphically in Figure S1. The most common DDIs that required changes in chronic therapy were observed in the psychiatric medication group in the PWID cohort.

The majority of participants were infected with G1a (155, 35%) and G3 (140, 32.4%), with PWIDs more frequently infected with G3, whereas non-PWIDs predominantly had G1b infection (*p* < 0.01). The highest proportion of participants were in the F0/1 fibrosis stage (200, 46.3%), while the F4 stage was more prevalent among non-PWIDs (*p* = 0.023). In the subset of patients with cirrhosis, PWIDs were significantly more likely to have Child–Pugh Class A, whereas non-PWIDs were predominantly classified as Class B or C (*p* < 0.001).

Hepatocellular carcinoma (HCC) was significantly more common in the non-PWID group (*p* < 0.001). A more detailed overview of the therapeutic and diagnostic modalities used in the study is provided in Table 2.

Individual analysis of the extracted subgroups revealed several statistically significant findings. In the problematic PWID group, there was a significantly higher proportion of males (*p* = 0.021), and participants were younger compared to other categories (*p* = 0.001). Additionally, problematic PWIDs had lower levels of liver fibrosis (*p* = 0.014) and fewer cirrhotic patients (*p* = 0.024) compared to the other subgroups. In the ex-PWID subgroup, participants were statistically significantly older (*p* = 0.041) and exhibited higher levels of liver fibrosis (*p* = 0.025) compared to the other subgroups. No statistically significant difference was observed in achieving SVR12 among the PWID subgroups.

Analysis of the LFU lost-to-follow-up group of patients revealed that, out of a total of 20 patients from the PWID group, 16 (80%) were from the problematic PWID subgroup, while 4 (20%) were from the ex-PWID subgroup. When considering the LFU lost-to-follow-up patients in the control group, it was observed that patients in the problematic PWID sub-group were statistically significantly more likely to be LFU lost to follow-up (*p* = 0.001). Of the total 24 patients LFU lost to follow-up, 12 were lost during therapy, with the majority being treated with the medication sofosbuvir/velpatasvir ± ribavirin for 12 weeks (8, 66.6%).

### 3.1. Predictors of Achieving SVR12—Intention to Treat (ITT)

The analysis of both Cox multivariate regression models in the non-PWID group revealed that younger age and a lower fibrosis stage were independently associated with higher rates of achieving SVR12. A detailed presentation is provided in Table 3.

The analysis of the three Cox multivariate regression models in the non-PWID group identified three factors independently associated with higher rates of achieving SVR12: a lower fibrosis stage, the absence of alcohol use disorder, and the absence of mood disorders. A detailed presentation is provided in Table 3 and Table 4.

### 3.2. Predictors of Achieving SVR12–Per Protocol (PP)

The analysis of three Cox multivariate regression models in the non-PWID group found that younger age and a lower fibrosis stage were independently associated with higher rates of achieving SVR12 in patients who were treated and monitored according to the treatment protocol. A detailed overview is provided in Table 5.

Additionally, the analysis of four Cox multivariate regression models in the non-PWID group identified three factors independently associated with higher rates of achieving SVR12: a lower fibrosis stage in patients treated and monitored according to the treatment protocol. A detailed presentation is available in Table 4 and Table 5.

The analyzed laboratory parameters recorded at the start of treatment did not show predictive significance for achieving SVR12 in both patient groups, as shown in Supplementary Table S2.

### 3.3. Factors Associated with Achieving SVR12 in PWID Subcategories

Variables with significant univariate associations with achieving SVR12 across PWID subcategories were included in the multivariate Cox regression model, with all models adjusted for sex and age. In Model 5, based on the ITT approach, a lower stage of fibrosis emerged as a predictor of achieving SVR12 among ex-PWIDs and problematic PWIDs, while the absence of mood disorders was identified as an additional predictor among problematic PWIDs. No significant predictors of SVR12 were identified for the PWIDs on OST subcategory. Similarly, Model 6, adjusted according to the PP approach, confirmed that a lower stage of fibrosis was predictive of SVR12 achievement among ex-PWIDs and problematic PWIDs. However, no predictors were observed for the PWIDs on OST subcategory in this model either. A detailed presentation is available in Table 6.

## 4. Discussion

It is estimated that the global prevalence of HCV infection is approximately 0.7%, corresponding to around 56.8 million people living with this infection at the beginning of 2020 [14]. Liver-related complications of CHC, including HCC and decompensated cirrhosis, are responsible for nearly 299,000 deaths per year [15]. DAAs represent a milestone in both clinical and epidemiological terms for HCV infection. Their high efficacy, short treatment duration, and minimal side effects have significantly improved patient outcomes, enabling high cure rates and reducing viral transmission within the population [16,17]. Regrettably, only 23% of individuals with CHC have been diagnosed, with merely 5% having received treatment [14].

Regardless of socioeconomic status, HCV is primarily transmitted through shared drug injection equipment, resulting in disproportionately high prevalence among PWIDs, ranging from 7.9% to 82% [2,18,19,20]. Harm reduction measures (primarily needle and syringe programs and opioid substitution therapy) and widespread treatment of PWIDs are essential for controlling CHC as a public health issue [21,22,23].

This study presents the outcomes of CHC treatment of PWIDs at the University Clinical Center in Serbia, which manages the highest number of patients in the country. The introduction of highly effective and well-tolerated, pangenotypic DAAs in 2022 has enabled the treatment of a significantly larger number of PWIDs compared to the previous period. The study included 432 patients treated over a two-year period (August 2022–August 2024) with DAAs in accordance with EASL recommendations. Of these, 163 (37.7%) PWIDs were classified as the study group, while the remaining 269 (62.3%) comprised the control group. The majority of PWIDs were ex-PWIDs on OST. The PWID group was significantly younger (45.74 ± 8.81 vs. 57.49 ± 14.57), with a significantly higher proportion of males compared to the control group (76.7% vs. 50.6%). PWIDs were less likely to have underlying comorbidities such as hypertension, diabetes mellitus, chronic kidney disease (including the need for dialysis), and malignant diseases, which can be explained by their younger age. According to the literature, PWIDs are expected to be younger compared to the general population [24]. On the contrary, mental health disorders (mood disorders, psychosis and neurosis) were more prevalent in the study group. This observation is consistent with data from literature. The high prevalence of psychiatric disorders among individuals with substance use disorder is widely acknowledged and thoroughly documented [25,26]. It is important to underline that psychiatric comorbidities among PWIDs are associated with poorer health-related outcomes [26]. Comorbidities were the primary factors determining which medications patient received, and these, in turn, influenced potential DDI with DDAs (psychiatric medications vs. antiarrhythmics, Figure S1).

Another important difference between PWIDs and non-PWIDs is the higher prevalence of alcohol use disorder (AUD) among those with substance use problems (*p* < 0.001). AUD has a profound impact on this population, given that it influences both prevention interventions and all stages of the HCV cascade of care, including diagnosis, linkage to care, treatment initiation, and the rate of sustained virologic response [27,28,29]. Not only does AUD contribute to liver disease itself, but it has also been recognized as a significant barrier to accessing DAA treatment [30,31]. Considering the higher risk of liver-related complications, patients with CHC and AUD should be prioritized for DAA treatment [32].

This study has demonstrated that HIV infection is significantly more common among PWIDs than among other patients with CHC (11.6% vs. 3.7%). This is not surprising, considering that the global prevalence of HIV infection is 20% among individuals with substance use disorders [33].

HCV genotype distribution was also impacted by drug abuse. Genotype 1 was the most prevalent, with subtype 1a predominating among PWIDs, whereas subtype 1b was characteristic of patients outside this group (*p* < 0.001). Consequently, EBR/GZR is more frequently administered to non-PWID populations. Liver cirrhosis was more prevalent in patients without a history of addiction. Serbian authors have already confirmed that subtype 1b was found in individuals older than 40 years, with an advanced stage of fibrosis and no history of intravenous drug use, characteristics that are consistent with those in this study [34]. Genotype 3 was the second most common in both analyzed groups, but it was significantly more frequently detected in PWIDs [34]. HCC was more common in non-PWIDs, which may be related to the fact that they were older than PWIDs and had a longer duration of infection prior to therapy [35].

The most pronounced disparity was observed in ITT SVR12 rates between PWIDs and non-PWIDs (85.3% vs. 97.8%), which can be attributed to a significantly higher LFU among PWIDs (*p* < 0.001). Ten PWID patients discontinued DAA therapy, and an additional 10 patients did not complete 12 weeks follow up period. In a recent German real-life study, 74% of 69 PWID patients achieved SVR 12, but in 20% of patients no information was available due to LFU [36]. The non-completion of therapy was seen among individuals treated with SOF/VEL, while all patients treated with G/P received full-length therapy, which could be explained by shorter duration of this therapeutic modality. Furthermore, more patients were lost during post treatment follow up in the SOF/VEL treatment arm. In the present study, 70 (42.9%) and 88 (54%) patients treated with SOF/VEL and G/P, respectively, voluntarily discontinued therapy. Given that the duration of therapy is defined, it might be considered to give priority to G/P therapy to PWIDs in cases without contraindications or significant DDI. On the other hand, follow up period might be a point which could be modified, thus enhancing the adherence to post treatment monitoring. Namely, recent research demonstrated that no significant difference was observed in the rates of SVR 4 and SVR 12 after pangenotypic DAA treatment which could serve as potential strategy for problematic PWIDs without OST [37]. Furthermore, the present study, along with other real-world data, highlights the importance of implementing OST for PWIDs, as most individuals LFU were problematic PWIDs not receiving OST [36]. High adherence to DAA as well as high SVR rates in PWIDs on OST were reported by Dore et al. [38]. Real-world data have also demonstrated no significant difference in SVR12 rates in PP analysis, irrespective of drug use history [39]. The results of our study are consistent with these findings, as high SVR12 rates were observed in both groups in the PP analysis (97.8% and 98.5%). In all patients treated PP, DAA adherence was over 95%, regardless of substance use disorder and AUD. Although AUD is common among PWIDs and some authors consider it a factor contributing to low adherence, there is also real-world experience that has not found this correlation [34,40]. Mental health disorders are an important factor influencing treatment adherence and contributing to LFU, suggesting that the treatment of CHC could be improved through collaboration with mental health and addiction specialists [30]. Achieving SVR resulted in an improvement in biochemical parameters, indicating that further disease progression was prevented [37]. Finally, advanced fibrosis was predictor of PP treatment failure among PWIDs, while mood disorders and AUL were associated with treatment interruptions before the scheduled completion time. Among non-PWIDs, older age and advanced fibrosis were identified as key predictors of PP treatment failure.

To the best of our knowledge, this study is the first to examine DAA treatment in PWIDs in Southeast Europe. The results provide detailed information on DAA treatment success and challenges in managing this sensitive population, highlighting the high risk of patients being LFU before completing treatment or achieving SVR12. This study has certain limitations. It was conducted at the University Clinical Center, where PWIDs were referred by addiction treatment facilities or general practitioners, as there is no active screening within this population, and treatment cannot be provided outside of university centers. This certainly has affected their numbers, proportion in the CHC population, duration of abstinence, and their motivation to seek treatment for HCV infection.

## 5. Conclusions

Due to the high prevalence of HCV, PWIDs are a key population for treatment in the effort to achieve the global elimination of HCV infection. The success of DAA therapy in this population does not differ from other CHC patients, provided that the treatment is conducted according to the protocol. However, treatment is associated with a higher risk of therapy interruption and higher LFU rates, especially in those diagnosed with AUD and psychiatric comorbidities. Problematic PWIDs without OST are especially vulnerable population, and are rarely included in the treatment and presented in the studies. However, this subgroup of patients might be the key one for achieving HCV elimination. Specifically, these patients have the highest chance of reinfection, which should not be a barrier to treatment and retreatment even though it is an objective issue.

All of the above highlights the need for a multidisciplinary approach in treating CHC in PWIDs, with the mandatory involvement of a psychiatrist and addiction specialist.

## Figures and Tables

**Table 1:** Overview of demographic characteristics, comorbidities, coinfections and substance use habits; CTD—connective tissue disease; n/a—not applicable.

Variable		PWID Cohort *n* = 163	Control Group *n* = 269	*p*
Age (mean ± SD)		45.74 ± 8.81	57.49 ± 14.57	0.001
Sex, *n* (%)	Male	125 (76.7%)	136 (50.6)	0.001
	Female	38 (24.3%)	133 (49.4)	
Chronic diseases, *n* (%)	Hypertension	24 (14.7%)	108 (40.1%)	0.001
	Other CV diseases	1 (0.6%)	20 (7.4%)	0.001
	Diabetes mellitus	9 (5.5%)	40 (14.9%)	0.003
	Respiratory diseases	9 (5.5%)	11 (4.1%)	0.328
	Chronic kidney failure	1 (0.6%)	22 (8.2%)	0.014
	Dialysis	1 (0.6%)	11 (4.1%)	0.036
	Malignant diseases	7 (4.3%)	48 (17.84%)	0.001
	CTD	3 (1.8%)	8 (3.0%)	0.125
	Neurological diseases	5 (3.1%)	11 (4.1%)	0.194
	Hypo/hyperthyroidism	4 (2.4%)	14 (5.2%)	0.216
	Mood disorders	8 (4.9%)	2 (0.7%)	0.001
	Psychoses/Neuroses	32 (19.6)	12 (4.5%)	0.001
	Alcohol use disorder	36 (22.1%)	18 (6.7%)	0.001
Coinfections, *n* (%)	HIV	19 (11.6%)	10 (3.7%)	0.021
	Antiretroviral therapy	17 (10.4%)	10 (3.7%)	0.102
	HBV	3 (1.8%)	5 (1.9)	0.264
	Resolved HBV	1 (0.6%)	1 (0.4%)	0.321
Drug use status *n* (%)	Ex-PWIDs	124 (76.1%)	n/a	
	Problematic PWIDs	39 (23.9%)		
	PWIDs on OST	96 (58.9%)		
	Active IV drug use	4 (2.4%)		
	Abstinence < 2 year	35 (21.5%)		
	Abstinence > 2 year	95 (58.3%)		
	Abstinence > 10 years	29 (17.8%)		
	Reported additional non-IV drug use	15 (9.2%)		
Substitution therapy *n* (%)	Without substitution	67 (41.1%)	n/a	
	Buprenorphine	51 (31.3%)		
	Methadone	45 (27.6%)		

**Table 2:** Evaluation of diagnostic and therapeutic characteristics and modalities in patient management; md—median; IQR—interquartile range; HCC—hepatocellular carcinoma.

Variable		PWID Cohort *n* = 163	Control Group *n* = 269	*p*
HCV RNA quantitative testing, median (IQR)		373,601.0 (70,225.0–1,385,121.2)	599,000.0 (147,000.0–2,110,000.0)	0.019
Sustained virologic response at post-treatment Week 12, *n* (%)		135 (82.2%)	259 (96.3%)	0.001
Hepatitis C genotype, *n* (%)	1a	66 (40.5%)	89 (34.3%)	0.042
	1b	10 (6.1%)	74 (28.6%)	0.001
	2	7 (4.3%)	12 (4.6%)	0.549
	3	65 (39.9%)	75 (29%)	0.021
	4	15 (9.2%)	19 (7.3%)	0.312
Antiviral therapy, *n* (%)	Glecaprevir/pibrentasvir	88 (54%)	107 (41.3%)	0.001
	Elbasvir/grazoprevir	5 (3.1%)	57 (22%)	
	Sofosbuvir/velpatasvir +/− ribavirin	70 (42.9%)	105 (40.5%)	
Method of liver fibrosis assessment, *n* (%)	Fibrosis-4 (FIB-4) index	59 (36.1%)	92 (35.5%)	0.197
	Liver stiffness measurement	93 (57.1%)	163 (62.9%)	
	Liver biopsy	34 (20.8%)	61 (23.5%)	
Fibrosis stage, *n* (%)	F0/1	69 (42.3%)	131 (50.6%)	0.087
	F2	32 (19.6%)	35 (13.5%)	0.031
	F3	18 (11%)	20 (7.7%)	0.058
	F4	44 (26.9%)	83 (32%)	0.023
Liver cirrhosis and Child–Pugh classification	Total cirrhotic patients	44 (26.9%)	83 (30.8%)	0.023
	Class A	35 (21.5%)	12 (4.4%)	0.001
	Class B	8 (4.9%)	65 (24.2%)	0.001
	Class C	1 (0.6%)	6 (2.2%)	0.026
Complications of liver cirrhosis	Ascites, n (%)	15 (9.2%)	35 (13.0%)	0.121
	Hepatic encephalopathy, *n* (%)	7 (4.3%)	8 (3.0%)	0.367
	Portal hypertension, *n* (%)	26 (15.9%)	34 (12.6%)	0.684
	Esophageal varices, *n* (%)	21 (12.8%)	33 (12.3%)	0.857
Total HCC patients		3 (1.8%)	20 (7.4%)	0.001

**Table 3:** Results of the Cox proportional hazard models: factors associated with SVR12—intention to treat; HR—hazard Ratio; CI—confidence interval; CTD—connective tissue disease; n/a—not applicable.

Model 1	PWID Cohort						Control Group					
Variable	Univariate			Multivariate			Univariate			Multivariate		
	HR	95% CI	*p*	HR	95% CI	*p*	HR	95% CI	*p*	HR	95% CI	*p*
Sex	0.587	0.60–1.15	0.531				1.002	0.78–1.28	0.989			
Ages	1.005	0.98–1.02	0.648				0.990	0.98–0.99	0.019	0.810	0.68–0.99	0.042
Stage of liver fibrosis	0.813	0.71–0.93	0.003	0.822	0.71–0.94	0.006	0.900	0.82–0.99	0.030	0.830	0.45–0.97	0.041
Hypertension	1.194	0.76–1.87	0.439				0.994	0.77–1.28	0.960			
Other CV diseases	n/a						1.044	0.65–1.67	0.857			
Diabetes mellitus	1.658	0.84–3.27	0.145	1.634	0.83–3.22	0.152	0.969	0.69–1.36	0.856			
Respiratory disease	0.835	0.39–1.79	0.644				0.934	0.50–1.73	0.828			
Chronic kidney failure	2.537	0.35–18.14	0.354				0.888	0.56–1.40	0.611			
Dialysis	2.554	0.36–18.25	0.350				1.024	0.54–1.93	0.942			
Malignant diseases	1.172	0.71–1.93	0.531				1.112	0.87–1.41	0.383			
CTD	0.935	0.13–6.69	0.946				0.742	0.36–1.53	0.416			
Neurological diseases	1.423	0.81–2.48	0.215				1.036	0.57–1.90	0.901			
Hypo/hyperthyroidism	1.333	0.42–4.20	0.623				1.611	0.93–2.77	0.086	1.749	0.97–3.03	0.158
Mood disorders	0.783	0.55–0.91	0.015	0.352	0.60–1.19	0.022	1.644	0.98–1.97	0.097			
Psychoses	0.251	0.15–1.14	0.174	0.325	0.22–1.49	0.189	1.023	0.97–1.15	0.250			
HIV	1.627	0.87–1.21	0.251				0.552	0.37–1.21	0.089			
Antiretroviral therapy	1.257	0.89–1.95	0.412				0.566	0.29–1.12	0.126			
HBV	1.681	0.81–2.68	0.851				0.810	0.31–1.23	0.187			
Resolved HBV	2.328	0.94–15.61	0.564				1.136	0.78–1.64	0.495			
Alcohol use disorder	0.657	0.44–0.99	0.046	0.691	0.46–1.05	0.045	0.790	0.46–1.35	0.790			
**Model 2**	**PWID Cohort**						**Control Group**					
**Variable**	**Univariate**			**Multivariate**			**Univariate**			**Multivariate**		
	**HR**	**95% CI**	***p***	**HR**	**95% CI**	***p***	**HR**	**95% CI**	***p***	**HR**	**95% CI**	***p***
Sex	0.587	0.60–1.15	0.531				1.002	0.78–1.28	0.989			
Ages	1.005	0.98–1.02	0.648				0.990	0.98–0.99	0.019			
Stage of liver fibrosis	0.813	0.71–0.93	0.003	0.815	0.57–1.16	0.259	0.900	0.82–0.99	0.030	0.854	0.74–0.98	0.188
HCV RNA quantitative	0.569	0.21–1.62	0.365				1.025	0.87–1.32	0.251			
Hepatitis C genotype	1.015	0.91–1.14	0.795				0.842	0.61–1.23	0.351			
Fibrosis-4 index	0.750	0.55–1.02	0.070	0.940	0.57–1.54	0.806	0.689	0.42–1.03	0.068	0.743	0.41–1.12	0.361
Child–Pugh class	1.124	0.61–2.08	0.709				1.236	0.87–2.41	0.257			
Degree of fibrosis kPa	0.981	0.95–1.01	0.241				0.874	0.69–1.20	0.364			
Degree of steatosis bD/m	0.998	0.99–1.00	0.473				0.987	0.94–1.05	0.255			
Advanced complications	0.806	0.37–1.75	0.584				0.451	0.21–1.07	0.178			
Hepatocellular carcinoma	0.934	0.30–2.94	0.907				0.887	0.51–1.25	0.361			

**Table 4:** Results of the Cox proportional hazard models: factors associated with SVR12 in PWID patients, ITT/PP; HR—hazard ratio; CI—confidence interval; CTD—connective tissue disease.

Model 3	Intention to Treat						Per Protocol					
Variable	Univariate			Multivariate			Univariate			Multivariate		
	HR	95% CI	*p*	HR	95% CI	*p*	HR	95% CI	*p*	HR	95% CI	*p*
Sex	0.587	0.60–1.15	0.531				0.625	0.51–1.18	0.241			
Ages	1.005	0.98–1.02	0.648				1.152	0.88–1.42	0.429			
Stage of liver fibrosis	0.813	0.71–0.93	0.003	0.822	0.71–0.94	0.006	0.751	0.62–0.97	0.014	0.698	0.28–1.12	0.136
Active IV drug use	0.952	0.81–1.08	0.352				0.962	0.91–1.02	0.598			
Abstinence < 1 year	0.852	0.74–1.01	0.152				0.789	0.61–1.28	0.345			
Abstinence > 1 year	1.061	0.98–1.15	0.362				0.958	0.84–1.14	0.129			
Abstinence > 10 years	1.098	0.99–1.19	0.120	1.120	0.98–1.25	0.562	1.108	0.98–1.12	0.108	1.217	0.98–2.25	0.651
Reported additional non-IV drug use	0.982	0.94–1.15	0.538				0.997	0.87–1.08	0.741			

**Table 5:** Results of the Cox proportional hazard models: factors associated with SVR12; per protocol. HR—hazard ratio; CI—confidence interval; CTD—connective tissue disease; n/a—not applicable.

Model 4	PWID Cohort						Control Group					
Variable	Univariate			Multivariate			Univariate			Multivariate		
	HR	95% CI	*p*	HR	95% CI	*p*	HR	95% CI	*p*	HR	95% CI	*p*
Sex	0.625	0.51–1.18	0.241				1.012	0.71–1.24	0.941			
Ages	1.152	0.88–1.42	0.429				0.910	0.85–0.99	0.002	0.954	0.90–1.18	0.152
Stage of liver fibrosis	0.751	0.62–0.970	0.014	0.612	0.16–0.87	0.001	0.937	0.81–0.99	0.024	0.758	0.41–0.84	0.012
Hypertension	0.691	0.21–1.19	0.284				0.990	0.79–1.18	0.231			
Other CV diseases	n/a						1.041	0.58–1.51	0.368			
Diabetes mellitus	1.126	0.62–2.11	0.610				0.952	0.62–1.35	0.195			
Respiratory disease	0.459	0.15–1.25	0.365				0.962	0.53–1.61	0.684			
Chronic kidney failure	1.985	0.39–9.14	0.287				0.842	0.51–1.45	0.012	0.569	0.11–0.84	0.021
Dialysis	1.854	0.28–9.10	0.921				1.039	0.24–1.99	0.121			
Malignant diseases	1.298	0.60–1.45	0.452				1.128	0.75–1.48	0.341			
CTD	0.541	0.27–5.15	0.859				0.765	0.32–1.58	0.356			
Neurological diseases	1.277	0.75–2.10	0.310				1.010	0.56–1.98	0.596			
Hypo/hyperthyroidism	1.201	0.39–3.58	0.341				1.602	0.90–2.17	0.198			
Mood disorders	0.672	0.17–1-15	0.187				1.664	0.98–1.97	0.329			
Psychoses	0.987	0.57–1.18	0.341				1.098	0.91–1.18	0.242			
HIV	1.974	0.98–2.85	0.490				0.685	0.31–1.23	0.095			
Antiretroviral therapy	1.657	0.86–2.58	0.697				0.106	0.25–1.28	0.362			
HBV	1.254	0.29–1.96	0.713				0.632	0.28–1.21	0.250			
Resolved HBV	1.964	0.91–3.69	0.946				1.058	0.58–1.32	0.541			
Alcohol use disorder	0.428	0.21–1.05	0.103	0.687	0.48–1.59	0.125	0.741	0.44–1.37	0.698			
**Model 5**	**PWID Cohort**						**Control Group**					
**Variable**	**Univariate**			**Multivariate**			**Univariate**			**Multivariate**		
	**HR**	**95% CI**	***p***	**HR**	**95% CI**	***p***	**HR**	**95% CI**	***p***	**HR**	**95% CI**	***p***
Sex	0.625	0.51–1.18	0.241				1.012	0.71–1.24	0.941			
Ages	1.152	0.88–1.42	0.429				0.910	0.90–0.99	0.002			
Stage of liver fibrosis	0.751	0.62–0.970	0.014	0.829	0.48–1.21	0.361	0.937	0.85–0.99	0.024			
HCV RNA quantitative	0.481	0.18–1.61	0.395				1.025	0.87–1.32	0.251			
Hepatitis C genotype	1.069	0.87–1.28	0.681				0.842	0.61–1.23	0.351			
Fibrosis-4 index	0.741	0.48–1.18	0.015	0.290	0.17–1.01	0.106	0.689	0.42–1.03	0.068	0.743	0.41–1.12	0.361
Child–Pugh class	1.292	0.58–1.98	0.691				1.236	0.87–2.41	0.257			
Degree of fibrosis kPa	0.920	0.87–1.56	0.108				0.874	0.69–1.20	0.364			
Degree of steatosis bD/m	0.816	0.49–1.27	0.397				0.987	0.94–1.05	0.255			
Advanced complications	0.895	0.31–1.12	0.512				0.451	0.21–1.07	0.178			
Hepatocellular carcinoma	0.845	0.26–2.43	0.592				0.887	0.51–1.25	0.361			

**Table 6:** Overview of multivariate regression models examining factors associated with achieving SVR12 among different categories of PWIDs, ITT/PP; HR—hazard ratio; CI—confidence interval; n/a—not applicable.

Model 5	Intention to Treat								
Variable	Ex-PWIDs			Problematic PWIDs			PWIDs on OST		
	HR	95% CI	*p*	HR	95% CI	*p*	HR	95% CI	*p*
Sex	0.552	0.21–1.02	0.425	0.671	0.32–1.01	0.624	0.694	0.31–1.02	0.197
Ages	1.015	0.91–1.03	0.563	1.210	0.89–1.54	0.512	1.154	0.81–1.47	0.910
Stage of liver fibrosis	0.746	0.62–0.98	0.043	0.822	0.71–0.94	0.006	0.695	0.42–1.02	0.120
Diabetes mellitus	0.658	0.18–1.24	0.252	n/a			n/a		
Mood disorders	0.610	0.14–1.18	0.268	0.785	0.36–0.96	0.026	0.789	0.61–1.28	0.345
Psychoses	0.590	0.28–1.50	0.910	n/a			0.842	0.60–1.02	0.236
Fibrosis-4 index	n/a			n/a			0.851	0.54–0.99	0.517
Child–Pugh class	0.691	0.99–1.19	0.561	n/a			n/a		
Reported additional non-IV drug use	n/a			n/a			0.997	0.87–1.08	0.741
**Model 6**	**Per Protocol**								
**Variable**	**Ex-PWIDs**			**Problematic PWIDs**			**PWIDs on OST**		
	**HR**	**95% CI**	***p***	**HR**	**95% CI**	***p***	**HR**	**95% CI**	***p***
Sex	0.684	0.45–1.01	0.679	0.781	0.34–1.16	0.245	0.611	0.29–1.06	0.180
Ages	1.011	0.90–1.03	0.829	1.087	0.97–1.12	0.621	1.255	0.73–1.79	0.360
Stage of liver fibrosis	0.654	0.51–0.83	0.028	0.822	0.71–0.94	0.030	0.741	0.54–1.21	0.152
Child–Pugh class	1.097	0.96–1.18	0.236	n/a			n/a		

## Data Availability

The data presented in this study are available on request from the corresponding authors. Data are unavailable due to privacy or ethical restrictions.

## Supplementary Materials

The following supporting information can be downloaded at: https://www.mdpi.com/article/10.3390/microorganisms12122554/s1, Figure S1: Overview of documented interactions between DAAs and chronic therapy medications in patients from both cohorts, recorded at the beginning of the follow-up period; Table S1: Presentation of laboratory parameters before and after DAA therapy in the PWID cohort and the control group. Legend: bolded values are statistically significant; Table S2: Presentation of Cox univariate and multivariate regression model analysis examining the predictive significance of laboratory parameters for achieving SVR12. HR—hazard ratio, CI—confidence interval, bolded values are statistically significant.
