# Supplementary material for: HCV Treatment Outcomes in PWID: Impact of Addiction History on SVR12
Source: Microorganisms. 2024 Dec 11;12(12):2554. doi: 10.3390/microorganisms12122554 (PMC11677223; doi:10.3390/microorganisms12122554)
Supplement: Supplementary file 1 [file microorganisms-12-02554-s001.zip › microorganisms-3341349-supplementary.pdf]

Supplementary Table S1. Presentation of laboratory parameters before and after DAA therapy in the PWID cohort and the control group. Legend: bolded values are statistically significant.

	PWID cohort N=163 n(%)			Control group N=269 (%)		
**Variable**	Pre-treatment	Post-treatment	*p*	Pre-treatment	Post-treatment	*p*
White blood cells	6.9 (5.1-8.4)	6.7 (5.3-9.0)	0.158	6.5 (5.2-8.1)	6.3 (5.1-8.0)	0.317
Neutrophils	3.7 (2.7-5.0)	3.9 (2.9-5.2)	0.045	3.7 (2.8-5.2)	3.7 (2.9-4.8)	0.740
Hemoglobin	142 (134-154)	144 (134-156)	0.601	138 (126-152)	137 (124-150)	0.986
MCV – mean corpuscular volume	89.6 (86.0-93.0)	90.2 (87,0-94.3)	**0.017**	90.1 (87.2-94.4)	91.0 (87.8-95.0)	0.226
Platelets	180 (129-230)	186 (135-238)	**0.007**	189 (140-233)	205 (143-249)	**0.012**
Gycemia	5.4 (4.9-6.4)	5.4 (5.1-6.1)	0.700	5.6 (5.0-7.0)	5.6 (4.9-6.4)	0.320
Urea	4.9 (4.0-6.3)	4.9 (4.0-6.0)	0.322	5.3 (4.1-7.7)	5.1 (4.0-6.9)	0.158
Creatinine	70.0 (82.0-92.0)	79.0 (67.0-88.2)	**0.007**	78 (66-96)	72 (60-85)	**<0.001**
Sodium	140.0 (137.0-141.0)	141.0 (139.0-143.0)	**0.002**	140 (138-142)	141 (139-143)	**0.007**
Potassium	4.2 (4.0-4.5)	4.1 (3.9-4.4)	0.999	4.3 (4.0-4.7)	4.3 (4.0-4.6)	0.156
Total bilirubine	10.2 (7.1-14.3)	9.1 (7.0-13.0)	0.414	10.1 (7.7-16.1)	9.0 (7.0-14.2)	0.258
Direct bilirubine	4.5 (3.5-6.6)	3.6 (2.8-4.7)	0.320	4.5 (3.3-7.6)	3.4 (2.6-5.0)	0.179
Aspartate aminotransferase	53 (31-82)	26 (18-40)	**<0.001**	51 (30-78)	25 (18-35)	**<0.001**
Alanine aminotransferase	73 (42-115)	34 (27-51)	**<0.001**	65 (39-107)	34 (25-51)	**<0.001**
Gamma glutamyl-transferase	59 (33-110)	39 (23-65)	**<0.001**	54 (30-94)	31 (22-50)	**<0.001**
Alkaline phosphateses	72 (62-100)	76 (56-93)	0.177	73 (53-95)	74 (54-91)	0.646
Proteins	69 (54-82)	78 (73-81)	**0.005**	70 (62-81)	79 (70-88)	**<0.001**
Albumin	40 (37-42)	40 (37-44)	0.468	40 (37-45)	40 (37-42)	0.315
INR – international normalized ratio	1.05 (1.01-1.15)	1.01 (0.99-1.04)	**0.008**	1.09 (1.00-1.25)	1.10 (1.01-1.23)	**0.045**
Prothrombine time (%)	90.0 (80.0-97.4)	99.3 (87.2-105.7)	**0.003**	89.2 (79.0-97.9)	86.8 (74.8-100.1)	**0.021**
Fibrinogene	2.6 (2.1-3.0)	2.6 (2.3-3.1)	0.319	2.5 (2.1-3.1)	2.8 (2.5-3.5)	0.657
Alpha-fetoprotein	3.8 (2.6-6.0)	3.0 (2.1-4.7)	**0.043**	4.5 (3.0-7.3)	3.2 (2.2-5.5)	**0.048**
Cholesterol	4.58 (3.90-5.13)	4.54 (4.10-5.12)	0.318	5.08 (4.20-6.00)	4.90 (4.23-5.82)	0.738
High density lipoprotein – HDL	2.1 (1.8-2.4)	2.6 (2.1-2.9)	0.127	2.4 (2.1-2.7)	2.8 (2.4-3.1)	0.089
Low density lipoprotein – LDL	2.5 (1.70-3.10)	2.4 (1.98-3.18)	0.083	2.87 (2.14-3.61)	2.80 (2.25-3.87)	0.315
Tryglicerides	1.25 (0.90-1.80)	1.34 (0.95-1.94)	0.423	1.45 (0.95-1.99)	1.37 (1.00-1.84)	0.334

Supplementary Table S2. Presentation of Cox univariate and multivariate regression model analysis examining the predictive significance of laboratory parameters for achieving SVR12. HR - hazard ratio, CI - confidence interval, bolded values are statistically significant.

Variable	Univariate			Multivariate			Univariate			Multivariate		
	HR	95% CI	p	HR	95%CI	p	HR	95%CI	P	HR	95%CI	p
Sex	0.587	0.60-1.15	0.531				1.002	0.78-1.28	0.989			
Ages	1.005	0.98-1.02	0.648				0.990	0.98-0.99	0.019			
Stage of liver fibrosis	0.813	0.71-0.93	**0.003**	0.912	0.80-0.99	**0.012**	0.900	0.82-0.99	0.030	0.964	0.89-0.99	**0.041**
White blood cells	0.856	0.62-1.25	0.129				1.081	1.06-1.10	0.823			
Neutrophils	1.156	0.59-1.84	0.608				1.848	0.74-2.46	0.930			
Hemoglobin	1.562	0.96-2.29	0.981				0.858	0.61-1.11	0.641			
MCV – mean corpuscular volume	1.740	0.64-3.50	0.436				2.821	0.98-4.51	0.732			
Platelets	1.859	0.97-2.85	0.241				1.990	1.97-2.01	0.013	1.082	0.97-1.14	0.187
Gycemia	0.841	0.78-1.03	0.320				0.948	0.91-1.02	0.197			
Urea	1.025	0.52-2.12	0.356				1.014	0.92-1.08	0.320			
Creatinine	0.951	0.81-1.25	0.910				0.880	0.61-1.01	0.788			
Sodium	1.510	0.83-2.25	0.916				1.013	0.92-1.10	0.897			
Potassium	0.978	0.72-1.31	0.883				1.001	0.82-1.14	0.320			
Total bilirubine	0.941	0.79-1.28	0.871				0.999	0.99-1.01	0.690			
Direct bilirubine	0.540	0.20-1.01	0.740				1.125	0.41-2.52	0.128			
Aspartate aminotransferase	0.941	0.90-1.26	0.620				0.905	0.77-1.06	0.397			
Alanine aminotransferase	0.651	0.21-0.98	**0.008**	0.820	0.62-1.02	0.120	0.748	0.33-1.66	0.474			
Gamma glutamyl-transferase	1.510	0.92-2.12	0.136				1.610	0.91-3.89	0.758			
Alkaline phosphateses	0.621	0.41-1.01	0.651				0.981	0.96-1.02	0.936			
Proteins	1.458	0.79-2.11	0.849				1.984	0.84-3.17	0.941			
Albumin	1.621	0.98-2.07	0.139				1.048	1.03-1.07	**0.019**	1.076	0.94-1.19	0.120
INR – international normalized ratio	0.987	0.92-1.06	0.817				1.031	0.91-1.05	0.422			
Prothrombine time (%)	1.541	0.99-1.91	0.364				1.008	0.99-1.01	0.620			
Fibrinogene	0.361	0.19-1.01	0.841				1.017	0.99-1.03	0.950			
Alpha-fetoprotein	0.462	0.11-0.86	**0.015**	0.682	0.41-1.03	0.251	0.970	0.94-0.99	0.036	0.834	0.45-1.12	0.260
Cholesterol	1.632	0.90-2.02	0.410				1.023	0.98-1.10	0.842			
High density lipoprotein – HDL	1.157	0.99-1.63	0.762				1.003	0.99-1.01	0.569			
Low density lipoprotein – LDL	0.584	0.12-1.01	0.371				1.002	0.99-1.01	0.185			
Tryglicerides	0.698	0.41-1.02	0.492				0.681	0.39-1.03	0.562			

		PWID cohort												Control group											
DAAs		SOF/VEL				ELB/GZR				G/P				SOF/VEL				ELB/GZR				G/P			
System	Class of medication	Do Not Coadminister	Potential Interaction	Potential Weak Interaction	No Interaction Expected	Do Not Coadminister	Potential Interaction	Potential Weak Interaction	No Interaction Expected	Do Not Coadminister	Potential Interaction	Potential Weak Interaction	No Interaction Expected	Do Not Coadminister	Potential Interaction	Potential Weak Interaction	No Interaction Expected	Do Not Coadminister	Potential Interaction	Potential Weak Interaction	No Interaction Expected	Do Not Coadminister	Potential Interaction	Potential Weak Interaction	No Interaction Expected
Cardiovascular	Calcium channel blocker	0	0	0	2	0	0	0	0	0	0	0	0	0	0	0	1	0	0	0	2	0	0	0	5
	ACE Inhibitors	0	0	0	5	0	0	0	1	0	0	0	6	0	0	0	25	0	0	0	5	0	0	0	16
	Diuretics	0	0	0	2	0	0	0	0	0	0	0	2	0	0	0	0	0	0	0	0	0	0	0	1
	Potassium-sparing diuretic	0	0	0	6	0	0	0	0	0	0	0	8	0	0	0	13	0	0	0	3	0	0	0	5
	Beta blockers	0	0	0	6	0	0	0	0	0	0	0	6	0	0	0	12	0	0	0	5	0	0	0	11
	Vasodilators	0	0	1	2	0	0	0	0	0	0	0	0	0	0	0	8	0	0	0	4	0	0	0	3
	Antiarrhythmics	1	0	0	0	0	0	0	0	1	0	0	0	0	1	0	0	1	0	0	0	2	0	0	0
Psychiatric	Antidepressants	0	0	1	2	0	0	0	2	0	0	1	2	0	0	1	2	0	0	0	2	0	0	0	2
	Anxiolytics	0	1	0	1	0	1	0	1	0	0	0	1	0	0	0	0	0	0	0	0	0	0	0	0
	Antipsychotics	0	0	0	1	0	0	1	0	0	1	0	2	0	0	0	1	0	0	0	2	0	0	0	1
	Mood Stabilizers	0	0	0	2	0	0	0	3	0	0	0	3	0	0	0	0	0	0	0	0	0	0	0	0
	Neuroleptics	0	0	1	0	0	0	0	0	0	0	0	0	0	0	0	0	0	0	0	0	0	0	0	0
	Stimulants	0	0	1	0	0	0	0	0	0	0	0	0	0	0	0	0	0	0	0	1	0	0	0	0
	Hypnotics and Sedatives	0	0	0	0	0	0	0	0	0	0	0	0	0	0	0	1	0	0	0	0	0	0	0	0
	Antiparkinsonian Medications	0	1	0	0	0	0	0	0	0	0	0	0	0	0	0	0	0	0	0	0	0	0	0	1
	Nootropics	0	0	0	1	0	0	0	0	0	0	0	1	0	0	0	1	0	0	0	0	0	0	0	0
	Antidepressant-Antipsychotic Combinations	0	0	0	0	0	0	0	1	0	0	0	1	0	0	0	0	0	0	0	0	0	0	0	0
	Methadone	0	0	0	15	0	0	0	19	0	0	0	11	n/a				n/a				n/a			
	Naltrexone	0	0	0	0	0	0	0	0	0	0	0	0												
	Buprenorphine/Naloxone	0	0	0	0	0	0	0	0	0	0	0	2												
	Buprenorphine	0	0	0	25	0	0	0	10	0	0	0	16												
Respiratory	Bronchodilators	0	0	5	0	0	0	0	2	0	0	0	0	0	0	4	0	0	0	0	2	0	0	3	0
	Inhaled corticosteroids	0	0	0	1	0	0	0	0	0	0	0	0	0	0	0	0	0	0	0	1	0	0	0	0
	Leukotriene Modifiers	0	0	0	0	0	0	0	0	0	0	0	0	0	0	0	1	0	0	0	0	0	0	0	0
	Antihistamines	0	0	0	0	0	0	0	0	0	0	0	0	0	0	0	0	0	0	0	1	0	0	0	1
	Mucolytics and Expectorants	0	0	0	0	0	0	0	0	0	0	0	0	0	0	0	0	0	0	0	0	0	0	0	0
	Immunomodulators	0	0	0	0	0	0	0	0	0	0	0	0	0	0	0	0	0	0	0	0	0	0	0	0
Rheumatologic	NSAIDs	0	0	0	1	0	0	0	1	0	0	0	0	0	0	0	4	0	0	0	3	0	0	0	0
	Disease-Modifying Anti-Rheumatic Drugs	0	0	1	0	0	0	0	0	0	0	0	0	0	0	0	0	0	0	0	1	0	0	0	0
	Antimalarials	0	0	0	0	0	0	0	0	0	0	0	0	0	0	0	0	0	0	0	0	0	0	0	0
	Immunosuppressive Drugs	0	0	0	1	0	0	0	0	0	0	0	0	0	0	0	0	0	0	0	0	0	0	0	0
	Phosphodiesterase Inhibitors	0	0	0	0	0	0	0	0	0	0	0	0	0	0	0	1	0	0	0	0	0	0	0	0
	Glucocorticoids	0	0	0	0	0	0	0	0	0	0	0	0	0	0	0	0	0	0	0	1	0	0	0	0
Endocrine	Insulins	0	0	0	0	0	0	0	0	0	0	0	0	0	0	0	0	0	0	0	0	0	0	0	0
	Oral Antidiabetic Drugs	0	0	0	0	0	0	0	0	0	0	0	0	0	0	0	0	0	0	0	0	0	0	0	0
	Thyroid Medications	0	0	0	0	0	0	0	0	0	0	0	0	0	0	0	0	0	0	0	0	0	0	0	0
	Medications for Calcium and Bone Metabolism	0	0	0	2	0	0	0	1	0	0	0	1	0	0	0	0	0	0	0	0	0	0	0	0
Antiviral drugs	Antiretroviral drugs	0	0	0	5	0	0	0	4	0	0	0	10	0	0	0	2	0	0	0	1	0	0	0	0
	Hepatitis B medications	0	0	0	1	0	0	0	2	0	0	0	0	0	0	0	7	0	0	0	1	0	0	0	2

Figure S1. Overview of documented interactions between DAAs and chronic therapy medications in patients from both cohorts, recorded at the beginning of the follow-up period.
